# Impact of distance education on academic performance in a pharmaceutical care course

**DOI:** 10.1371/journal.pone.0175117

**Published:** 2017-04-06

**Authors:** Agnes Nogueira Gossenheimer, Tamires Bem, Mára Lucia Fernandes Carneiro, Mauro Silveira de Castro

**Affiliations:** 1 Federal University of Rio Grande do Sul, School of Pharmacy, Porto Alegre, Rio Grande do Sul, Brazil; 2 Federal University of Rio Grande do Sul, Institute of Psychology of the, Porto Alegre, Rio Grande do Sul, Brazil; Universidade do Porto, Faculdade de Farmácia, PORTUGAL

## Abstract

The objective of this study was to compare the performance of pharmacy students from a Pharmaceutical Care course, taught in both distance education (DE) and campus-based formats using active methodologies. For two semesters, students (n = 82) taking the course studied half the subject in the distance education format and half in person. Questionnaires were applied at the beginning of the semester aimed to outline the demographic profile of the students. Their grade in the course was evaluated to determine their performance. The Module 1 (Information on Medication) average on the campus-based was 7.1225 and on DE was 7.5519, (p = 0.117). The Module 2 (Pharmaceutical Services) average on the campus-based was 7.1595 and on distance education was 7.7025, (p = 0.027*). There was a difference in learning outcomes in the Pharmaceutical Care Course between face-to-face and distant education. Therefore, the student performance was better in the distance education module, indicating distance education can be satisfactorily used in Pharmacy Programs.

## Introduction

In light of the recent use of virtual modes in health education, few studies that researched whether distance classes show differences in academic performance in relation to the campus-based format, for the same course and conditions were found [1].

The advantages of teaching by way of distance classes are often readily apparent, particularly with regard to student access and availability, but there are some drawbacks. Students cannot develop the socialization and interpersonal skills that normally accompany traditional learning methods. For pharmacy students, the daily interaction with faculty and peers to aid in the development of professionalism can also be lost. Although there was no difference in student outcomes between distance and presential classes for a variety of higher education programs, there is little data describing [2–5] the effects of technology in the pharmacy curricula [6–7].

In 2010, Harrison et al. determined that there were 20 schools with courses using distance education in the USA, including 16 campuses running in parallel, resulting in separate student groups for all four years of the PharmD program. Of these 16 schools, 12 delivered content synchronously, 1 school delivered content asynchronously, and 3 schools delivered content in a hybrid of both synchronous and asynchronous formats [8]. This continuous and substantial growth illustrates the importance of understanding the potential impact on academic performance of the students' experience in distance education. Some studies show that distance education had a positive impact, like Creighton University, where distance students performed better than students on campus. The authors concluded that distance students were not hindered by the delivery method [9]. On the other hand, Reid and colleagues showed the delivery method of a course does not correlate with academic performance when they compared the academic data of PharmD students at the traditional campus versus distance campuses of the University of Florida College of Pharmacy [10].

Research has identified that cognitive factors such as learning experiences, academic performance and distance class formats are comparable to those observed for campus-based classes; [11–13] however, the perception and satisfaction levels of distance education professors and students has not shown the same consistence [14, 15]. Factors such as accessibility to materials, interaction between students and professors, time management and expense may all influence the opinions of distance education participants [16].

The Federal University of Rio Grande do Sul (UFRGS) has a policy of evaluating its courses. Possible formats for courses are campus-based, distance and blended (a combination of campus-based and distance), with a maximum of 20% of a given course taught using the distance format. The current evaluation method aims to determine how the student is doing in the different learning formats. All courses at the university are evaluated each semester by the students. As a result of these available new approaches the Undergraduate Committee of the School of Pharmacy agreed to develop an additional evaluation to verify the effects of the changes made. This initiative was part of the institutional quality control program.

The Pharmaceutical Care II course became part of the undergraduate studies program at the School of Pharmacy at UFRGS—Federal University of Rio Grande do Sul—in 2008, and is currently taught in a mixed format that includes DE in its teaching program.

In this respect, the debate on evaluating Pharmaceutical Care courses [17], an innovative field in the pharmaceutical program [18], is a significant one, as is assessing the implementation of formats such as DE and active teaching methodologies in the School of Pharmacy [19]. Mesquita et. al. evaluated the performance of students before and after the pharmaceutical care course. They mentioned a study limitation: since the active learning approach was not compared to a traditional teaching methodology, it cannot be determined whether the former is the superior approach for the teaching of pharmaceutical care.

The aim was to compare the performance of pharmacy students in the course taught using distance and campus-based classes.

## Materials and methods

### Description of the pharmaceutical care II course

Content in the pharmaceutical care II course is taught using both the campus-based and distance education formats. A portion of the content was offered in the distance learning format and the remainder as face-to-face (campus-based) classes, comprising two separate learning modules. This modular content was taught in both formats and with the same learning objectives.

The Table 1 presents classes held in the course and how each activity was offered in distance or face-to-face modalities. The distance modality took place in the Moodle platform, where didactic materials and learning objects were posted, as well as the activities were accomplishmed. It was also offered in each class the possibility of solving doubts via asynchronous forum. The face-to-face modality was held in the classroom or in a computer lab, as needed. The face-to-face classes also had the teaching materials available on the Moodle platform. Both in the distance and in the classroom, written texts and scientific articles were made available by teachers, helping the students to complement their studies.

**Table 1:** Comparison of the topics covered in the discipline of pharmaceutical care II, in the distance and face-to-face modalities.

CONTENTS OF THE CLASS	DISTANCE EDUCATION	FACE-TO-FACE`EDUCATION
Lesson 1—Module 1. Presentation.	Presentation of the course: objectives, contents, form of evaluation. How to use moodle platform features. How to be a virtual student.	Presentation of the course: objectives, contents, form of evaluation.
Lesson 2—Module 1: Information and rational use of medicines.	Virtual visit to the Medicines Information Center; Individual led study posted on the Moodle platform. Medication Information Center Discussion Forum.	Face-to-face visit to the Medication Information Center; Individual directed study and face-to-face discussion.
Lesson 3—Module 1: Passive and Active Information on Medications.	Search tutorial on drug information sites asynchronously assisted. Exercise presented in the form of games about drug information sources.	Web site search tutorial, presented in a computer lab, with exercises on the topic.
Lesson 4—Module 1: Sources of Medication Information.	Asynchronous recorded lesson on book presentation and tutorial on the MICROMEDEX database. Exercise on information search.	Classes about books and presentation of the MICROMEDEX database. Exercise on information search.
Lesson 5—Module 1: Primary sources.	Asynchronous recorded classroom on Structures of scientific articles and Introduction to critical reading. Critical Analysis Exercise of article posted on the platform.	Lecture on Structures of scientific articles and Introduction to critical reading. Critical Analysis Exercise of an article made, delivered and presented in class.
Lesson 1- Module 2: Pharmaceutical Care in the World and DRC 44.	Recorded asynchronous class on concepts and context of pharmaceutical attention and Brazilian legislation on the subject. Exercise on legislation applied to professional practice. Reminder about virtual student.	Lecture on concepts and context of pharmaceutical care and Brazilian legislation on the subject. Exercise on legislation applied to professional practice.
Lesson 2- Module 2: Dispensing	Reading text about Dispensing medications. Videos Analysis of dispensing simulations and posting of evaluations in the Moodle platform. Beginning of the development of a drug dispensing roadmap, using the knowledge obtained in module 1.	Lecture on dispensing medications. Projection of videos of simulations of dispensation with evaluation exercise. Beginning of the development of a drug dispensing roadmap, using the knowledge obtained in module 1.
Lesson 3—Module 2: Treatment adherence	Reading of book chapter and articles on the topic. Development of a conceptual map to be posted on the moodle platform.	Expositive-dialogue session on adherence to treatment. Discussion on the topic with the preparation of a script about the problems of adherence to treatment.
Lesson 4—Module 2: Medication Errors	Court of the Jury synchronous on the platform Moodle, using the discussion forum, on a case of medication error.	Court of the Jury on a case of medication error.
Lesson 5—Module 2: Distribution System of Medicines in Hospitals and Blood Pressure Measurement	Video asynchronous lecture about the distribution system of medicines in hospitals and on the measurement of blood pressure.	Lecture about the distribution system of medicines in hospitals and practical demonstration on the measurement of blood pressure.
Lesson 6- Module 2: Pharmaceutical Guidance	Text, video-oriented pharmaceutical guidance, audio simulation. Exercise of registration of attendance and posting in the platform. Submission of the final version of the drug dispensing roadmap that will be simulated in the skills assessment.	Lecture about orientation, presentation of simulation of attendance and exercise of registration of attendance. Submission of the final version of the drug dispensing roadmap that will be simulated in the skills assessment.
Lesson 7—Module 2: Gymkhana	Gymkhana content review asynchronously via Moodle.	Gymkhana review of classroom content.
Lesson 8—Module 2: service simulation.	Presential assessment of skills in patient care: simulation of individually recorded care.	Presential assessment of skills in patient care: simulation of individually recorded care.
Lesson 9—Module 2: patient care simulation.	Presential assessment of skills in patient care: simulation of individually recorded care.	Presential assessment of skills in patient care: simulation of individually recorded care.
Lesson 10—General Test.	Knowledge test on the contents of the 2 modules.	Knowledge test on the contents of the 2 modules.

The first module deals with Drug Information, addressing types of information and search strategies related to drugs, with students analyzing medical prescriptions to be used in simulated care in the second module. The second module addresses methods of caring for patients, including dispensing medication and pharmacist counseling, as well as factors that interfere in outcomes, such as medication errors and adherence to pharmacological treatment.

The present study used course data relating to students and assessments of the course for 2012 first and second semesters. In the 2012 first semester, the drug information module was face-to-face and second module was DE. In the 2012 second semester it was the opposite. Thus, all students enrolled in the course had a module in each modality, face-to-face and distance.

Regardless of the setting, participants were taught the same classes and content, by the same professors and with identical assessment objectives. The only difference between the two groups was the learning format (distance or campus-based). For students in the classroom mode the Moodle platform was used as a repository of the classes and the same didactic materials available to the students of the distance modality. In this way, there are no disparities in the process of consulting the teaching materials.

Participants filled out knowledge and performance evaluations during the semester, which included the following content:

Module 1: Exercise assessing the ability to compile information medication plus participation in classes;
Module 2: Introducing basic theory for dispensing medication and pharmaceutical counseling; evaluating videos depicting dispensing procedures and pharmaceutical counseling; trial by jury, where students are divided into groups representing the defense, prosecution and jury and use their technical knowledge to analyze an actual case involving medication error reported in the media; simulations of dispensing medications; scavenger hunts using knowledge gained in the course and participation in classes;

The evaluation of module I was composed by the evaluation of exercises developed in each class and an individual assignment on evaluation of a prescription, handed out at the end of the Module. On the other hand, the evaluation of module II was composed by the average score of Gymkhana, Jury's Court and Simulation of attendance (an activity recorded face-to-face at the end of the module, developing communication skills).

In addition to the notes per module, at the end of the semester the students carried out the same face-to-face test, with written questions, to evaluate the content learned. The evaluation included closed and open questions and was based on the resolution of clinical cases, encompassing the contents of the two modules. All evaluations were corrected in duplicates, by the teacher of the discipline and by the teaching trainee, without blindness. When there was disagreement, the consensus was sought and the basis of the evaluation was discussed.

### Sample

Because this study is an institutional control of quality of the introduction of the new modality—DE—in the curriculum, the sample was composed of students enrolled in the 4^th^ phase of the undergraduate pharmacy program at the Federal University of Rio Grande do Sul, in 2012 first (n = 40) and second (n = 42) semesters, who were taking the Pharmaceutical Care II course.

The 40 students who enrolled in the Pharmacy Major the first semester of 2012 and the 42 students enrolled in the second semester of 2012 attended the course, being Module I in the distance modality and Module 2 in the face-to-face modality.

### Instruments

The instruments used in this quality control study consisted of an assessment questionnaire, applied at the beginning of the semester to identify student profiles and preferences. The questionnaires were structured based on items found in comparative studies regarding the DE and campus-based formats, researched beforehand in a review published studies.

### Student profile

The survey applied at the beginning of the course aimed to outline the demographic profile of the students and their level of digital inclusion, while considering baseline variables. These variables may be related to student perception about DE and campus-based formats, as well as their performance.

### Academic performance assessment

In order to compare the academic performance of students in distance or campus-based activities, the grade for each module was analyzed using both modes and the final result for the course. The grades ranged from 0 to 10, with 7 being the approval minimum mark.

### Statistical analysis and ethical aspects

Data were analyzed using version 17.0 version of SPSS software. The student’s t-test, paired t-test, ANOVA, Mann Whitney U and Pearson’s correlation were applied for statistical comparisons in questionnaire 1, when appropriate.

The Wilcoxon test was used to analyze the differences between the DE and campus-based formats for the different aspects investigated in blocks 1 and 2 of questionnaire 2. Data with ordinal variables was analyzed using the non-parametric Mann Whitney U test (to compare class assessments between the two semesters, addressed by questions in block 4 of questionnaire 2).

The study was approved by Graduation Committee of Undergraduate Pharmacy Program at the Federal University of Rio Grande do Sul, as part of course evaluation. The consent was verbal, since it was part of the evaluation of the discipline, as explained to the students in the first day of class. The Ethics Committee of the University, when consulted, stated that because it is the evaluation of the course, it would not be necessary to sign a written consent form. The graduation committee approved this procedure, after consultation with the ethics committee.

## Results

### Student profile

In the 2012 first semester forty students were enrolled in the course, with forty-two registered for 2012 second semester. Seventy-four students answered the questionnaire, because four of those enrolled withdrew from the course and four were not present during the application.

The profile of students from the 2012 year is shown in Table 2. The questionnaire also evaluated the students' level of digital knowledge, with no differences between groups, with 23% who already had attended distance classes. (See Table 2).

**Table 2:** Profile of students from the pharmaceutical care II course, in semesters 01 and 02 of 2012.

Variables	N	%
**Students Enrolled (respondents)**		
First semester	40 (36)	48,78 (37,22)
Second semester	42 (38)	51,21 (46,34)
**Gender**		
Female	66	89,29
Male	8	10,71
**Age**		
Average (years)	23.9 (19–31)	
**How do you prefer to work?**		
Groups	16	22,5
Double	38	53,5
Individual	17	24
**Pharmacy area you want to specialize (open question)**		
Industrial Pharmacy	14	20,6
Clinical analysis	12	17,6
Research	4	5,9
Hospital pharmacy	4	5,9
Teaching	4	5,9
Cosmetology	4	5,9
Criminal Expertise	3	4,4
Do not know	16	23,5
Others	6	10,3
**Satisfaction with the Pharmacy course**		
Completely Dissatisfied	0	0
Somewhat Satisfied	11	15.1
Satisfied	52	71.2
Very Satisfied	7	9.6
Completely Satisfied	3	4.1
**Advantages of distance education**		
Comfort, no need to leave home	25	39,7
Ease and speed in performing tasks	9	14,3
Time flexibility	8	12,7
Tools contribute to learning	7	11,1
Others	14	22,2
**Disadvantages of distance education**		
Lack of contact between students	15	34,1
Difficulty of solving doubts	10	22,7
Incomplete understanding, does not capture as much	6	13,6
There are no disadvantages	4	9,1
Others	9	20,5
**Have you used the MOODLE platform yet?**		
Yes	74	100
**How often do you check emails?**		
Every day	73	98,6
Once a week	1	1,4
**Where from do you access internet?**		
From home	68	93,2
From work	2	2,7
From university	1	1,4
From several places	2	2,7
**Have you taken an DE course before?**		
Yes	23	31.1
No	51	68.9

### Student performance

Student performance was assessed using the scores for each module and the final examination grade, comparing classes for the two formats by means of overall exam averages. The objective was to determine if there were significant differences in the performance of students between formats. As demonstrated in Table 3, there were no significant differences between the course modules.

**Table 3:** Average student's grade in the modules of the discipline of pharmaceutical care II.

	Módulo I/ Module I	Módulo II/Module II	Total
**Average grade**	7,3288	7,4416	7,5364

A comparison of the average performance from semester to semester according to the learning format used produced the data shown in Table 4. For module 1, which addressed Drug Information, there were no significant differences between the two formats, although the grade achieved for distance mode was higher than the campus-based format. In Module II, regarding the pharmaceutical services, there was a significant difference, and the average of the students was higher in the distance modality, being 7.7025.

**Table 4:** Comparison of average grades for modules I and II of the pharmaceutical care II course.

	N	Average	*P*^1^.
Performance in Module 1			
(campus-based)	40	7.1225	0.117
(distance)	37	7.5519	
Performance in Module 2			
(distance)	40	7.7025	0.027*
(campus-based)	37	7.1595	

^1^ The t-test (*p<0.05) was used to compare average grades for modules I and II in each semester

## Discussion

Findings regarding student performance when learning via distance or campus-based classes using active methodologies may be influenced by several factors [20]. Most students reported they were satisfied with the Pharmacy Program and that their expectations were consistent, in part, with that discussed in the course. However, most areas of expertise that students intended to follow were different from those addressed in the course, with a quarter of students still undecided. Studies should be developed to evaluate if motivation for a professional area or indecision can influence student performance.

With respect to the level of digital inclusion, students displayed significant affinity, checking their emails daily and capable of accessing the platform from home with the knowledge to complete the tasks set. These data are in line with the generation to which they belong, where digital inclusion forms a substantial part of their daily routine. Known as Generation Y, these individuals were born from the early 1980s to the early 2000s and have been graduating and entering the work market in recent years, marking the beginning of a new form of influence on society. They are characterized as individuals adept at multi-tasking; seeking recognition for what they do and often requesting feedback on their work; aiming at establishing informal relationships, valuing flexibility and convenience; adopting individual behaviors, stimulated by technological ease and with a broader spectrum of relationships, aided by social networks [21]. These aspects are reflected in the advantages attributed to DE expressed at the beginning of the course, but contrast against the loss of some elements of the in-person (campus-based) communication process.

The point most highlighted as an advantage of DE using the Internet was that of convenience, allowing students to study without leaving home. The item most commonly cited as a disadvantage of DE was the lack of contact between students and the difficulty in resolving queries. This point may have arisen because student’s previous experiences with DE prior to beginning the Pharmaceutical Care course caused them to associate the virtual environment with content that does not value interaction or tutoring that allows queries to be resolved online. The fact that students thought there was less support and interaction in DE is due not only to course formats, but also to the profile of students who are not used to taking DE classes with this type of interaction.

Research by Fainholc refers to mediators as human and non-human communication proposals that allow a person, group or organization to either completely or partially perform the functions of support, assistance and negotiation using different support systems [22]. As such, it is by focusing on these mediating points, both when preparing professors and tutors and improving the activities used, that the course should be continually improved, with the goal of making DE a closer reflection of reality and full with social and cultural meaning.

Halaban [23] also discusses interaction in distance education, analyzing reconfiguration of habits and depleting of interactions in present-daily life of contemporary societies. This phenomenon is based on the expansion of digital networks and the use of technology by individuals, and can be used to explain the fact that students have listed the lack of interaction the greatest disadvantage of distance education, since social interactions are also reduced. Another key point identified by Fainholc are mediations, which involve cultural critical reflection on the multiple heterogeneity and temporality of mediations as a primordial space or bond recreating personal or group meanings within a globally interconnected world. In the present study, mediations occurred synchronously and asynchronously mediate by the computer via the Internet, such dialogues taking place in course discussion forums [22]. Of course, these mediations are consistent with how students interact with their classmates in the University setting and in society. As such, the lack of interaction cited by the students themselves likely reflects how they interact with the world.

As pointed out by Guadagnin [24], one of the challenges of distance education at the pedagogical level is to create a spirit of community, as the intensification of the interaction between people who have affinities of interests tends to foster the dissemination and generation of knowledge in the virtual community. According to Fainholc, it is important to redesign, reflect, rethink and revise the communication process mediated by digital means in order to involve, recognize and integrate the focal point with new forms [22]. It is precisely this reinvention that presents a challenge, since the novel is new both for those who create and those who learn. Carnevale [25] found that students in distance learning seek out many characteristics of face-to-face mode, including interaction with the teacher, with colleagues and the community environment created in the classroom.

Students receiving their pharmacy education via distance education pathway scored higher compared with students receiving their pharmacy education via the traditional face-to-face pathway. This indicates that distance classes are receiving at least an equivalent curricular experience compared to that received by face-to-face. Our data indicate that learning occurred; that students were able to demonstrate competency of the abilities. The explanation for that performance be higher in the distance education may be related to the fact that students have more support material in distance education, has more autonomy to manage their study time and to conduct a preliminary study to class. On the other hand, it is evident that in the distance classes the student is obliged to participate more effectively, since the moodle platform allows to verify if the student has accessed the contents, how many times he has done it and if he has actually accomplished the tasks assigned. Carr [26] found that students enrolled in a psychology course performed better in distance education, but were less satisfied with this modality. In Carr’s [26] reaserch, distance learning students had a grade average 5% higher than face-to-face students, but with less satisfaction.

Distance education presented advantages over face-to-face, as students had a higher performance, but this result was only statistically significant in module 2. The fact that students learned more or similarly allows on to state that this type of modality is effective and can be used satisfactorily in pharmaceutical education. It is important to point out that both in the distance and face-to-face modality, the students had the same content, the same form of evaluation and the same opportunity to revise the contents, since even in the face-to-face modality the equal right to consult the didactic material was ensured. One factor that may explain the best performance in the distance mode is the greater obligation to pay attention to the contents. It is impossible to verify whether the student in the classroom is paying attention to the contents, even in some active learning tasks.

It can also be related that the advantages of distance education pointed out by students, as regarded to convenience and the possibility of studying at the most appropriate time, may also have been a factor that influenced academic performance.

## Conclusions

The use of campus-based or distance modes, applying active methodologies, showed differences with respect to the acceptance of students in the Pharmaceutical Care II Course. Student performance was better in DE modules, which may be related to the requirement for greater participation during the semester.

## Supporting information

S1 Questionnaire Questionnaire 1. (DOCX)

S2 Questionnaire Questionnaire 2. (DOC)
