# Supplementary material for: Impact of distance education on academic performance in a pharmaceutical care course
Source: PLoS One. 2017 Apr 6;12(4):e0175117. doi: 10.1371/journal.pone.0175117 (PMC5383158; doi:10.1371/journal.pone.0175117)
Supplement: S1 Questionnaire — (DOCX) [file pone.0175117.s001.docx]

**Questionário – 2012/02**

Olá Prezado(a) aluno(a)!!

Este questionário visa obter informações básicas sobre seus conhecimentos e habilidades em informática, para direcionar melhor os esforços e conteúdos da Disciplina de Atenção Farmacêutica II. Este questionário também tem como objetivo conhecer melhor o aluno da disciplina.

1. Nome Completo (favor preencher com letras maiúsculas):

1. E-mail (favor preencher com letras maiúsculas seu e-mail):
2. Sexo: F( ) M ( )
3. Idade: __________
4. Estado Civil:

( )Casado/Mora junto ( )Solteiro ( ) Separado/ Divorciado ( )Viúvo ( ) Outros

1. Qual sua ocupação

( ) Estagiário IC ( ) Empregado assalariado ( ) Profissional liberal ( ) Empresário

( ) Aposentado ( ) Dona de casa ( ) Estagiário Extensão ( ) Estagiário voluntário

( ) Outras? ____________________________________________________________

1. Tem filhos?

Não ( ) Sim ( ). Quantos?_______

1. Como prefere fazer trabalhos? ( ) Em grupo ( ) Em dupla ( ) Individualmente
2. Que área pretende seguir na Farmácia:
3. Como está sua satisfação em relação ao curso de Farmácia até agora?

( ) Nada satisfeito

( ) Pouco Satisfeito

( ) Satisfeito

( ) Muito satisfeito

( ) Totalmente satisfeito

1. Qual a sua expectativa em relação à Disciplina de Atenção Farmacêutica II?
2. Já repetiu na Faculdade alguma vez? Se sim, quantas vezes, quais disciplinas e por quê?
3. Quantas disciplinas está cursando neste semestre, contando a Atenção Farmacêutica II?
4. Em qual escola cursou seu segundo grau? Particular ou Pública?

ATENÇÃO: Seja absolutamente sincero(a) ao preencher este questionário. Marque apenas uma opção de cada item.

1. Você gosta de trabalhar com computadores?

A - [ ] sim

B - [ ] razoavelmente

C - [ ] não

D - [ ] não sei. Poucas vezes ou nunca tive oportunidade de trabalhar com computadores.

1. Qual é o local onde você acessa mais à Internet?

A - [ ] desde casa

B - [ ] desde o trabalho

C - [ ] desde a universidade

D - [ ] desde um Lan House, Cybercafe, etc.

E - [ ] desde vários deles

F - [ ] desde nenhum deles, até agora.

1. Você tem um endereço de e-mail próprio (que é só para seu uso pessoal)?

A - [ ] Sim B - [ ] Não

1. Há quanto tempo que você usa e-mail? A - [ ] Fiz um agora para o curso B - [ ] já tenho faz de 1 a 3 meses C - [ ] entre 4 e 6 meses D - [ ] há mais de 6 meses que tenho e-mail.
2. Com qual freqüência você costuma verificar seu e-mail aproximadamente? A - [ ] todos os dias ou várias vezes por semana B - [ ] uma vez por semana C - [ ] uma vez cada quinze dias D - [ ] uma vez por mês ou menos
3. Já fez algum outra disciplina a distância ou semipresencial?

A - [ ] sim

B -[ ] não

1. Você participa de alguma comunidade virtual ou rede social (tipo Orkut, Facebook ou similares)? A - [ ]sim B - [ ] não C - [ ] Nem sei o que é isso. Qual(is)? _________________________________________
2. Você sabe preencher dados em uma planilha de Excel? A - [ ] Sim. Já fiz isso várias vezes B - [ ] Mais ou menos C - [ ] Não D - [ ] Nem sei o que é isso.
3. Você sabe fazer cálculos e estatísticas básicas com os dados de uma planilha de Excel? A - [ ] Sim. Já fiz isso várias vezes B - [ ] Mais ou menos C - [ ] Não D - [ ] Nem sei o que é isso
4. Você já utilizou a Plataforma Moole?
5. Você encontrou alguma dificuldade na utilização da Plataforma Moodle? Se sim, quais?
6. Qual a sua percepção a respeito do ensino à distância? Vantagens e desvantagens?

**Questionnaire – 2012/02**

Greetings dear student!

This questionnaire aims to find out about your basic IT knowledge and skills in order to better guide the content of the Pharmaceutical Attention II course. This form will also help us to get to know our students better.

1. Full name (please use capital letters):

1. E-mail (please use capital letters):
2. Sex : F ( ) M ( )
3. Age: __________
4. Marital status:

( ) Married/ Living together ( ) Single ( ) Separated/ Divorced ( ) Widowed ( ) Other

1. Profession:

( ) Academic research intern ( ) Salaried worker ( ) Liberal professional ( ) Executive

( ) Retired ( ) Housewife ( ) University extension intern ( ) Volunteer intern

( ) Other ____________________________________________________________

1. Do you have children?

No ( ) Yes ( ). How many ?_______

1. How do you prefer to work? ( ) As part of a team ( ) With a colleague ( ) Individually
2. What field of pharmacy do you intend to work in?:
3. What is your level of satisfaction regarding the pharmacy course so far?

( ) Not satisfied at all

( ) Slightly satisfied

( ) Satisfied

( ) Very satisfeid

( ) Totally satisfied

1. Describe your level of expectation regarding the Pharmaceutical Attention II course?
2. Have you ever taken a university course over again? If so, how many times, which courses and for what reason?
3. How many courses are you taking this semester including Pharmaceutical Attention II?
4. What high school did you attend? Was it a public or private school?

ATTENTION: Please be totally sincere as you complete this questionnaire. Mark only one answer for each question.

1. Do you like to work on computers?

A - [ ] yes

B - [ ] somewhat

C - [ ] no

D - [ ] don't know. I have little or no opportunity to work on computers.

1. Where do you access the internet most often?

A - [ ] from home

B - [ ] from work

C - [ ] from the university

D - [ ] from a Lan house or Cybercafés

E - [ ] from a variety of places

F - [ ] none of these, so far

1. Do you have your own e-mail address (solely for personal use)?

A - [ ] Yes B - [ ] No

1. How long have you been using e-mail? A - [ ] I got an address just now for the course. B - [ ] I've used e-mail for 1 to 3 months. C - [ ] for 4 to 6 months D - [ ] more than 6 months
2. Approximately how often do you check your e-mail? A - [ ] every day or a few times a week B - [ ] once a week C - [ ] twice a month D - [ ] once a month or less
3. Have you taken any other distance or part-time attendance course?

A - [ ] yes

B -[ ] no

1. Are you part of any virtual community or social network (such as Orkut, Facebook or the like)? A- [ ] yes B - [ ] no C - [ ] I am not familiar with them.
2. Do you know how to fill in an Excel spreadsheet? A - [ ] Yes. I've done so a few times. B - [ ] More or less. C - [ ] No. D - [ ] I don't know what that is.
3. Do you know how to do calculations and basic statistics with the data on an Excel spreadsheet? A - [ ] Yes. I've done so a few times. B - [ ] More or less. C - [ ] No. D - [ ] I don't know what that is.
4. Have you ever used a Moodle platform?
5. Did you have any problems using a Moodle platform? If so, what problems?
6. What do you think of distance learning? What are the advantages and disadvantages?
