# Supplementary material for: Impact of distance education on academic performance in a pharmaceutical care course
Source: PLoS One. 2017 Apr 6;12(4):e0175117. doi: 10.1371/journal.pone.0175117 (PMC5383158; doi:10.1371/journal.pone.0175117)
Supplement: S2 Questionnaire — (DOC) [file pone.0175117.s002.doc]

**Avaliação das Aulas – 2012/02**

Este questionário visa saber qual foi a sua percepção e avaliação em relação às aulas da Disciplina de Atenção Farmacêutica II.

1. Nome Completo (com letras maiúsculas):

1. Como prefere fazer trabalhos em aula? ( ) Em grupo ( ) Em dupla ( ) Individualmente
2. Como é seu grau de satisfação em relação ao curso de Farmácia até agora?

( ) Nada satisfeito ( ) Pouco Satisfeito ( ) Satisfeito ( ) Muito satisfeito ( ) Totalmente satisfeito

**BLOCO 1 – AVALIAÇÃO DO MÓDULO I – EAD**

4- A seguir são listadas algumas afirmações sobre a modalidade EAD, gostaríamos que você dissesse, com base na sua experiência e/ou opinião, se concorda ou discorda de cada uma delas, considerando uma escala que varia entre 1 (discordo totalmente) a 5 (concordo totalmente). SC0 (Sem Condições de Opinar, caso tenha faltado a aula).

| **O Módulo I EAD...** | **GRAU DE CONCORDÂNCIA** | | | | | **SCO** |
| --- | --- | --- | --- | --- | --- | --- |
|  | **Discordo totalmente** | **Discordo** | **Nem concordo, nem discordo** | **Concordo** | **Concordo totalmente** |  |
| ... possibilita aprendizagem | ( 1 ) | ( 2 ) | ( 3 ) | ( 4 ) | ( 5 ) | ( 9 ) |
| ... estimula a participação do aluno | ( 1 ) | ( 2 ) | ( 3 ) | ( 4 ) | ( 5 ) | ( 9 ) |
| ... proporciona ao aluno um pensamento reflexivo | ( 1 ) | ( 2 ) | ( 3 ) | ( 4 ) | ( 5 ) | ( 9 ) |
| ... proporciona interação entre os colegas | ( 1 ) | ( 2 ) | ( 3 ) | ( 4 ) | ( 5 ) | ( 9 ) |
| ... é inovador | ( 1 ) | ( 2 ) | ( 3 ) | ( 4 ) | ( 5 ) | ( 9 ) |
| ...permite autonomia no aprendizado | ( 1 ) | ( 2 ) | ( 3 ) | ( 4 ) | ( 5 ) | ( 9 ) |
| ... permite flexibilidade de tempo | ( 1 ) | ( 2 ) | ( 3 ) | ( 4 ) | ( 5 ) | ( 9 ) |
| ... proporciona apoio pedagógico aos alunos | ( 1 ) | ( 2 ) | ( 3 ) | ( 4 ) | ( 5 ) | ( 9 ) |
| ... condiz com a sua preferência de aprendizagem | ( 1 ) | ( 2 ) | ( 3 ) | ( 4 ) | ( 5 ) | ( 9 ) |
| ... estimula o desenvolvimento de habilidades | ( 1 ) | ( 2 ) | ( 3 ) | ( 4 ) | ( 5 ) | ( 9 ) |
| ...possui aceitação dos alunos | ( 1 ) | ( 2 ) | ( 3 ) | ( 4 ) | ( 5 ) | ( 9 ) |

5- Qual o seu grau de satisfação geral com relação ao Módulo I EAD?

( ) Muito insatisfeito ( ) Insatisfeito ( ) Satisfeito ( ) Muito satisfeito

Considerações adicionais:___________________________________________________________________________________________

__________________________________________________________________________________________________________

**BLOCO 2 – AVALIAÇÃO DO MÓDULO II – PRESENCIAL**

6- A seguir são listadas algumas afirmações sobre a modalidade PRESENCIAL, gostaríamos que você dissesse, com base na sua experiência e/ou opinião, se concorda ou discorda de cada uma delas, considerando uma escala que varia entre 1 (discordo totalmente) a 5 (concordo totalmente). SC0 (Sem Condições de Opinar).

| O **Módulo II em EAD...** | **GRAU DE CONCORDÂNCIA** | | | | | **SCO** |
| --- | --- | --- | --- | --- | --- | --- |
|  | **Discordo totalmente** | **Discordo** | **Nem concordo, nem discordo** | **Concordo** | **Concordo totalmente** |  |
| ... possibilita aprendizagem | ( 1 ) | ( 2 ) | ( 3 ) | ( 4 ) | ( 5 ) | ( 9 ) |
| ... estimula a participação do aluno | ( 1 ) | ( 2 ) | ( 3 ) | ( 4 ) | ( 5 ) | ( 9 ) |
| ... proporciona ao aluno um pensamento reflexivo | ( 1 ) | ( 2 ) | ( 3 ) | ( 4 ) | ( 5 ) | ( 9 ) |
| ... proporciona interação entre os colegas | ( 1 ) | ( 2 ) | ( 3 ) | ( 4 ) | ( 5 ) | ( 9 ) |
| ... é inovadora | ( 1 ) | ( 2 ) | ( 3 ) | ( 4 ) | ( 5 ) | ( 9 ) |
| ...permite autonomia no aprendizado | ( 1 ) | ( 2 ) | ( 3 ) | ( 4 ) | ( 5 ) | ( 9 ) |
| ... permite flexibilidade de tempo | ( 1 ) | ( 2 ) | ( 3 ) | ( 4 ) | ( 5 ) | ( 9 ) |
| ... proporciona apoio pedagógico aos alunos | ( 1 ) | ( 2 ) | ( 3 ) | ( 4 ) | ( 5 ) | ( 9 ) |
| ...condiz com a sua preferência de aprendizagem | ( 1 ) | ( 2 ) | ( 3 ) | ( 4 ) | ( 5 ) | ( 9 ) |
| ... estimula o desenvolvimento de habilidades | ( 1 ) | ( 2 ) | ( 3 ) | ( 4 ) | ( 5 ) | ( 9 ) |
| ...possui aceitação dos alunos | ( 1 ) | ( 2 ) | ( 3 ) | ( 4 ) | ( 5 ) | ( 9 ) |

1. Qual o seu grau de satisfação geral com relação ao Módulo II PRESENCIAL?

( ) Muito insatisfeito ( ) Insatisfeito ( ) Satisfeito ( ) Muito satisfeito

Considerações adicionais:__________________________________________________________________________________________

__________________________________________________________________________________________________________

**BLOCO 3 – AVALIAÇÃO ENTRE PRESENCIAL X EAD:**

8. A seguir são listados na tabela alguns itens relacionados à aula e ao aprendizado. Você deve relacionar à modalidade que preferir, EAD ou PRESENCIAL, marcando um “X” em apenas uma opção:

| Critério | EAD | PRESENCIAL |
| --- | --- | --- |
| Proporciona mais tempo de estudo desta disciplina |  |  |
| Beneficia a relação aluno professor |  |  |
| Beneficia a relação aluno faculdade |  |  |
| Maior satisfação em relação às aulas |  |  |
| Exigência maior |  |  |
| Maior aprendizagem |  |  |
| Melhor qualidade |  |  |

**BLOCO 4 – AVALIAÇÃO DE CADA AULA DO MÓDULO I:**

AULA 1 – Apresentação da disciplina e do EAD.

Considerações gerais:___________________________________________________________________

AULA 2- A informação e o uso racional sobre medicamentos. (visita virtual ao CIM e estudo dirigido)

| **AULA 2** | **AVALIAÇÃO GERAL** | | | | **SCO** |
| --- | --- | --- | --- | --- | --- |
|  | **Péssima** | **Ruim** | **Boa** | **Ótima** |  |
| Metodologia de aula | ( 1 ) | ( 2 ) | ( 3 ) | ( 4 ) | ( 9 ) |
| Material de Apoio | ( 1 ) | ( 2 ) | ( 3 ) | ( 4 ) | ( 9 ) |
| Tarefas | ( 1 ) | ( 2 ) | ( 3 ) | ( 4 ) | ( 9 ) |

Considerações gerais:___________________________________________________________________

AULA 3- Informação Passiva e Ativa. (tutorial de busca em sites e exercícios sobre fontes)

| **AULA 3** | **AVALIAÇÃO GERAL** | | | | **SCO** |
| --- | --- | --- | --- | --- | --- |
|  | **Péssima** | **Ruim** | **Boa** | **Ótima** |  |
| Metodologia de aula | ( 1 ) | ( 2 ) | ( 3 ) | ( 4 ) | ( 9 ) |
| Material de Apoio | ( 1 ) | ( 2 ) | ( 3 ) | ( 4 ) | ( 9 ) |
| Tarefas | ( 1 ) | ( 2 ) | ( 3 ) | ( 4 ) | ( 9 ) |

Considerações gerais:___________________________________________________________________

AULA 4- Fontes de informação sobre Medicamentos.(Apresentação de livros e do MICROMEDEX).

| **AULA 4** | **AVALIAÇÃO GERAL** | | | | **SCO** |
| --- | --- | --- | --- | --- | --- |
|  | **Péssima** | **Ruim** | **Boa** | **Ótima** |  |
| Metodologia de aula | ( 1 ) | ( 2 ) | ( 3 ) | ( 4 ) | ( 9 ) |
| Material de Apoio | ( 1 ) | ( 2 ) | ( 3 ) | ( 4 ) | ( 9 ) |
| Tarefas | ( 1 ) | ( 2 ) | ( 3 ) | ( 4 ) | ( 9 ) |

Considerações gerais:___________________________________________________________________

AULA 5- Fontes primárias. (Estruturas de artigos científicos/ Introdução à leitura crítica/ Trabalho de Análise Crítica de Artigo)

| **AULA 5** | **AVALIAÇÃO GERAL** | | | | **SCO** |
| --- | --- | --- | --- | --- | --- |
|  | **Péssima** | **Ruim** | **Boa** | **Ótima** |  |
| Metodologia de aula | ( 1 ) | ( 2 ) | ( 3 ) | ( 4 ) | ( 9 ) |
| Material de Apoio | ( 1 ) | ( 2 ) | ( 3 ) | ( 4 ) | ( 9 ) |
| Tarefas | ( 1 ) | ( 2 ) | ( 3 ) | ( 4 ) | ( 9 ) |

Considerações gerais:___________________________________________________________________

AULA 6- Apresentação do Trabalho da Receita

| **AULA 6** | **AVALIAÇÃO GERAL** | | | | **SCO** |
| --- | --- | --- | --- | --- | --- |
|  | **Péssima** | **Ruim** | **Boa** | **Ótima** |  |
| Metodologia de aula | ( 1 ) | ( 2 ) | ( 3 ) | ( 4 ) | ( 9 ) |
| Material de Apoio | ( 1 ) | ( 2 ) | ( 3 ) | ( 4 ) | ( 9 ) |
| Tarefas | ( 1 ) | ( 2 ) | ( 3 ) | ( 4 ) | ( 9 ) |

Considerações gerais:___________________________________________________________________

**BLOCO 5 – AVALIAÇÃO DE CADA AULA DO MÓDULO II:**

AULA 7- Atenção Farmacêutica no Mundo e RDC 44

| **AULA 7** | **AVALIAÇÃO GERAL** | | | | **SCO** |
| --- | --- | --- | --- | --- | --- |
|  | **Péssima** | **Ruim** | **Boa** | **Ótima** |  |
| Metodologia de aula | ( 1 ) | ( 2 ) | ( 3 ) | ( 4 ) | ( 9 ) |
| Material de Apoio | ( 1 ) | ( 2 ) | ( 3 ) | ( 4 ) | ( 9 ) |
| Tarefas | ( 1 ) | ( 2 ) | ( 3 ) | ( 4 ) | ( 9 ) |

Considerações gerais:___________________________________________________________________

AULA 8- Dispensação (Análise dos Vídeos sobre dispensação)

| **AULA 8** | **AVALIAÇÃO GERAL** | | | | **SCO** |
| --- | --- | --- | --- | --- | --- |
|  | **Péssima** | **Ruim** | **Boa** | **Ótima** |  |
| Metodologia de aula | ( 1 ) | ( 2 ) | ( 3 ) | ( 4 ) | ( 9 ) |
| Material de Apoio | ( 1 ) | ( 2 ) | ( 3 ) | ( 4 ) | ( 9 ) |
| Tarefas | ( 1 ) | ( 2 ) | ( 3 ) | ( 4 ) | ( 9 ) |

Considerações gerais:___________________________________________________________________

AULA 8- Adesão ao tratamento (Intervenções em grupo e elaboração do roteiro)

| **AULA 8** | **AVALIAÇÃO GERAL** | | | | **SCO** |
| --- | --- | --- | --- | --- | --- |
|  | **Péssima** | **Ruim** | **Boa** | **Ótima** |  |
| Metodologia de aula | ( 1 ) | ( 2 ) | ( 3 ) | ( 4 ) | ( 9 ) |
| Material de Apoio | ( 1 ) | ( 2 ) | ( 3 ) | ( 4 ) | ( 9 ) |
| Tarefas | ( 1 ) | ( 2 ) | ( 3 ) | ( 4 ) | ( 9 ) |

Considerações gerais:___________________________________________________________________

AULA 9 - Erros de medicação (Tribunal)

| **AULA 9** | **AVALIAÇÃO GERAL** | | | | **SCO** |
| --- | --- | --- | --- | --- | --- |
|  | **Péssima** | **Ruim** | **Boa** | **Ótima** |  |
| Metodologia de aula | ( 1 ) | ( 2 ) | ( 3 ) | ( 4 ) | ( 9 ) |
| Material de Apoio | ( 1 ) | ( 2 ) | ( 3 ) | ( 4 ) | ( 9 ) |
| Tarefas | ( 1 ) | ( 2 ) | ( 3 ) | ( 4 ) | ( 9 ) |

Considerações gerais:___________________________________________________________________

AULA 10- Sistema de Distribuição de medicamentos e Medida da PA

| **AULA 10** | **AVALIAÇÃO GERAL** | | | | **SCO** |
| --- | --- | --- | --- | --- | --- |
|  | **Péssima** | **Ruim** | **Boa** | **Ótima** |  |
| Metodologia de aula | ( 1 ) | ( 2 ) | ( 3 ) | ( 4 ) | ( 9 ) |
| Material de Apoio | ( 1 ) | ( 2 ) | ( 3 ) | ( 4 ) | ( 9 ) |
| Tarefas | ( 1 ) | ( 2 ) | ( 3 ) | ( 4 ) | ( 9 ) |

Considerações gerais:___________________________________________________________________

AULA 11- Orientação Farmacêutica

| **AULA 11** | **AVALIAÇÃO GERAL** | | | | **SCO** |
| --- | --- | --- | --- | --- | --- |
|  | **Péssima** | **Ruim** | **Boa** | **Ótima** |  |
| Metodologia de aula | ( 1 ) | ( 2 ) | ( 3 ) | ( 4 ) | ( 9 ) |
| Material de Apoio | ( 1 ) | ( 2 ) | ( 3 ) | ( 4 ) | ( 9 ) |
| Tarefas | ( 1 ) | ( 2 ) | ( 3 ) | ( 4 ) | ( 9 ) |

Considerações gerais:___________________________________________________________________

AULA 12- Gincana Atenfar

| **AULA 12** | **AVALIAÇÃO GERAL** | | | | **SCO** |
| --- | --- | --- | --- | --- | --- |
|  | **Péssima** | **Ruim** | **Boa** | **Ótima** |  |
| Metodologia de aula | ( 1 ) | ( 2 ) | ( 3 ) | ( 4 ) | ( 9 ) |
| Material de Apoio | ( 1 ) | ( 2 ) | ( 3 ) | ( 4 ) | ( 9 ) |
| Tarefas | ( 1 ) | ( 2 ) | ( 3 ) | ( 4 ) | ( 9 ) |

Considerações gerais:___________________________________________________________________

AULA 13- Simulação da Orientação (Gravação)

| **AULA 13** | **AVALIAÇÃO GERAL** | | | | **SCO** |
| --- | --- | --- | --- | --- | --- |
|  | **Péssima** | **Ruim** | **Boa** | **Ótima** |  |
| Metodologia de aula | ( 1 ) | ( 2 ) | ( 3 ) | ( 4 ) | ( 9 ) |
| Material de Apoio | ( 1 ) | ( 2 ) | ( 3 ) | ( 4 ) | ( 9 ) |
| Tarefas | ( 1 ) | ( 2 ) | ( 3 ) | ( 4 ) | ( 9 ) |

Considerações gerais:___________________________________________________________________

**BLOCO 6- AUTO-AVALIAÇÃO DO ALUNO:**

| **Em relação a sua atitude em relação a disciplina de ATENFAR II....** | **GRAU DE CONCORDÂNCIA** | | | | | SCO |
| --- | --- | --- | --- | --- | --- | --- |
|  | Discordo totalmente | Discordo | Nem concordo, nem discordo | Concordo | Concordo totalmente |  |
| ... acho que não me dediquei o máximo que poderia. | ( 1 ) | ( 2 ) | ( 3 ) | ( 4 ) | ( 5 ) | ( 9 ) |
| ... não me adaptei a tecnologia utilizada | ( 1 ) | ( 2 ) | ( 3 ) | ( 4 ) | ( 5 ) | ( 9 ) |
| ... não me interesso pela disciplina | ( 1 ) | ( 2 ) | ( 3 ) | ( 4 ) | ( 5 ) | ( 9 ) |
| ... foi a primeira vez que tive aula em EAD | ( 1 ) | ( 2 ) | ( 3 ) | ( 4 ) | ( 5 ) | ( 9 ) |
| ... preparo-me para as aulas lendo os textos sugeridos | ( 1 ) | ( 2 ) | ( 3 ) | ( 4 ) | ( 5 ) | ( 9 ) |
| ... mantenho uma atitude atenta e participativa | ( 1 ) | ( 2 ) | ( 3 ) | ( 4 ) | ( 5 ) | ( 9 ) |
| ... após a aula consolido o que aprendi | ( 1 ) | ( 2 ) | ( 3 ) | ( 4 ) | ( 5 ) | ( 9 ) |
| ...aprendo mais com leituras de textos | ( 1 ) | ( 2 ) | ( 3 ) | ( 4 ) | ( 5 ) | ( 9 ) |
| ...aprendo mais com métodos ativos | ( 1 ) | ( 2 ) | ( 3 ) | ( 4 ) | ( 5 ) | ( 9 ) |

**BLOCO 7- SATISFAÇÃO EM RELAÇÃO À ATENÇÃO FARMACÊUTICA II**

| **Em relação à disciplina de Atenção Farmacêutica II...** | **GRAU DE CONCORDÂNCIA** | | | | | SCO |
| --- | --- | --- | --- | --- | --- | --- |
|  | Discordo totalmente | Discordo | Nem concordo, nem discordo | Concordo | Concordo totalmente |  |
| ... acho que fica melhor quando é dada em EAD | ( 1 ) | ( 2 ) | ( 3 ) | ( 4 ) | ( 5 ) | ( 9 ) |
| ... é bem planejada | ( 1 ) | ( 2 ) | ( 3 ) | ( 4 ) | ( 5 ) | ( 9 ) |
| ... é estimulante | ( 1 ) | ( 2 ) | ( 3 ) | ( 4 ) | ( 5 ) | ( 9 ) |
| ... é relevante para o curso de Farmácia | ( 1 ) | ( 2 ) | ( 3 ) | ( 4 ) | ( 5 ) | ( 9 ) |
| ...possui um conteúdo adequado | ( 1 ) | ( 2 ) | ( 3 ) | ( 4 ) | ( 5 ) | ( 9 ) |
| ...é importante para a minha formação profissional | ( 1 ) | ( 2 ) | ( 3 ) | ( 4 ) | ( 5 ) | ( 9 ) |
| ...teve seus objetivos alcançados | ( 1 ) | ( 2 ) | ( 3 ) | ( 4 ) | ( 5 ) | ( 9 ) |

Críticas e Sugestões:___________________________________________________________________________________________

_______________________________________________________________________________________________________

_______________________________________________________________________________________________________

**Evaluation of the Classes – 2012/02**

This questionnaire aims to learn your opinion and evaluation of the Pharmaceutical Attention II course classes.m

1. Full name (use capital letters):

1. How do you prefer to do classroom tasks? ( ) In a group ( ) In pairs ( ) Individually
2. How would you rate your level of satisfaction with regards to the Pharmacy course so far?

( ) Not satisfied at all. ( ) Slightly satisfied. ( ) Satisfied. ( ) Very satisfied. ( ) Totally satisfied.

**PART 1 – EVALUATION OF MODULE I – DL**

4- Following there is a list of statements regarding the Distance Learning mode. Could you tell us if you agree or disagree with each one based on your experience and/or opinion? Rate them on a scale from 1 (totally disagree) to 5 (totally agree) And SCO (unable to give an opinion, if you failed to attend a class).

| **Module I DL ...** | **Range of Agreement** | | | | | **SCO** |
| --- | --- | --- | --- | --- | --- | --- |
|  | **Totally disagree** | **Disagree** | **Neither agree nor disagree** | **Agree** | **Totally agree** |  |
| ... enables learning | ( 1 ) | ( 2 ) | ( 3 ) | ( 4 ) | ( 5 ) | ( 9 ) |
| ...promotes student participation | ( 1 ) | ( 2 ) | ( 3 ) | ( 4 ) | ( 5 ) | ( 9 ) |
| ... provides student with food for thought | ( 1 ) | ( 2 ) | ( 3 ) | ( 4 ) | ( 5 ) | ( 9 ) |
| ... provides interaction among colleagues | ( 1 ) | ( 2 ) | ( 3 ) | ( 4 ) | ( 5 ) | ( 9 ) |
| ... is innovative | ( 1 ) | ( 2 ) | ( 3 ) | ( 4 ) | ( 5 ) | ( 9 ) |
| ... enables learning autonomy | ( 1 ) | ( 2 ) | ( 3 ) | ( 4 ) | ( 5 ) | ( 9 ) |
| ... allows flexible hours | ( 1 ) | ( 2 ) | ( 3 ) | ( 4 ) | ( 5 ) | ( 9 ) |
| ... provides students with pedagogical support | ( 1 ) | ( 2 ) | ( 3 ) | ( 4 ) | ( 5 ) | ( 9 ) |
| ... agrees with your preferred style of learning | ( 1 ) | ( 2 ) | ( 3 ) | ( 4 ) | ( 5 ) | ( 9 ) |
| ... promotes skills development | ( 1 ) | ( 2 ) | ( 3 ) | ( 4 ) | ( 5 ) | ( 9 ) |
| ... is accepted by the students | ( 1 ) | ( 2 ) | ( 3 ) | ( 4 ) | ( 5 ) | ( 9 ) |

5- What is your level of satisfaction with Module I DL?

( ) Very unsatisfied ( ) Unsatisfied ( ) Satisfied ( ) Very satisfied

Additional comments::___________________________________________________________________________________________

__________________________________________________________________________________________________________

**PART 2 – EVALUATION OF MODULE II – CLASSROOM**

6- Following there is a list of statements regarding the traditional CLASSROOM or FACE-TO-FACE module. Could you tell us if you agree or disagree with each one based on your experience and/or opinion? Rate them on a scale from 1(totally disagree) to 5 (totally agree.And SCO (Unable to give an opinion, if you failed to attend a class).

| **Module II ...** | **RANGE OF AGREEMENT** | | | | | **SCO** |
| --- | --- | --- | --- | --- | --- | --- |
|  | **Totally disagree** | **Disagree** | **Neither agree nor disagree** | **Agree** | **Totally agree** |  |
| ... enables learning | ( 1 ) | ( 2 ) | ( 3 ) | ( 4 ) | ( 5 ) | ( 9 ) |
| ... promotes student participation | ( 1 ) | ( 2 ) | ( 3 ) | ( 4 ) | ( 5 ) | ( 9 ) |
| ...provides student with food for thought | ( 1 ) | ( 2 ) | ( 3 ) | ( 4 ) | ( 5 ) | ( 9 ) |
| ...provides interaction among colleagues | ( 1 ) | ( 2 ) | ( 3 ) | ( 4 ) | ( 5 ) | ( 9 ) |
| ...is innovative | ( 1 ) | ( 2 ) | ( 3 ) | ( 4 ) | ( 5 ) | ( 9 ) |
| ...permite autonomia no aprendizado | ( 1 ) | ( 2 ) | ( 3 ) | ( 4 ) | ( 5 ) | ( 9 ) |
| ...enables learning autonomy | ( 1 ) | ( 2 ) | ( 3 ) | ( 4 ) | ( 5 ) | ( 9 ) |
| ... provides students with pedagogical support | ( 1 ) | ( 2 ) | ( 3 ) | ( 4 ) | ( 5 ) | ( 9 ) |
| ...agrees with your preferred style of learning | ( 1 ) | ( 2 ) | ( 3 ) | ( 4 ) | ( 5 ) | ( 9 ) |
| ... promotes skills development | ( 1 ) | ( 2 ) | ( 3 ) | ( 4 ) | ( 5 ) | ( 9 ) |
| ...accepted by the students | ( 1 ) | ( 2 ) | ( 3 ) | ( 4 ) | ( 5 ) | ( 9 ) |

1. What is your level of satisfaction with Module II CLASSROOM?

( ) Very unsatisfied ( ) Unsatisfied ( ) Satisfied ( ) Very satisfied

Additional comments :__________________________________________________________________________________________

__________________________________________________________________________________________________________

**PART 3- COMPARISON BETWEEN CLASSROOM AND DL:**

8. Following there is a list of items regarding classes and learning. Select either DL or CLASSROOM as your preferred mode. Mark only one option with an “X” .

| Criteria | DL | CLASSROOM |
| --- | --- | --- |
| Provides more time to study this course |  |  |
| Benefits the student/teacher relationship |  |  |
| Benefits the student/university relationship |  |  |
| Greater satisfaction regarding classes |  |  |
| More demanding |  |  |
| Greater learning |  |  |
| Higher quality |  |  |

**PART 4 – EVALUATION OF EACH CLASS IN MODULE I:**

1st CLASS – Introduction to the course and to DL.

General opinion :___________________________________________________________________

2nd CLASS - Information and the rational use of medication. (virtual visit to the Medication Information Center and directed study)

| **2nd CLASS** | **GENERAL EVALUATION** | | | | **SCO** |
| --- | --- | --- | --- | --- | --- |
|  | **Awful** | **Bad** | **Good** | **Great** |  |
| Metodology of the class | ( 1 ) | ( 2 ) | ( 3 ) | ( 4 ) | ( 9 ) |
| Supporting materials | ( 1 ) | ( 2 ) | ( 3 ) | ( 4 ) | ( 9 ) |
| Tasks | ( 1 ) | ( 2 ) | ( 3 ) | ( 4 ) | ( 9 ) |

General opinion :___________________________________________________________________

3rd CLASS- Active and Passive Information. (tutorial on searching sites and exercises on sources)

| **3rd CLASS** | **GENERAL EVALUATION** | | | | **SCO** |
| --- | --- | --- | --- | --- | --- |
|  | **Awful** | **Bad** | **Good** | **Great** |  |
| Methodology of the class | ( 1 ) | ( 2 ) | ( 3 ) | ( 4 ) | ( 9 ) |
| Supporting materials | ( 1 ) | ( 2 ) | ( 3 ) | ( 4 ) | ( 9 ) |
| Tasks | ( 1 ) | ( 2 ) | ( 3 ) | ( 4 ) | ( 9 ) |

General opinion :___________________________________________________________________

4th CLASS- Sources of information on medication .(The books and MICROMEDEX are presented.)

| **4th CLASS** | **GENERAL EVALUATION** | | | | **SCO** |
| --- | --- | --- | --- | --- | --- |
|  | **Awful** | **Bad** | **Good** | **Great** |  |
| Methodology of the class | ( 1 ) | ( 2 ) | ( 3 ) | ( 4 ) | ( 9 ) |
| Supporting materials | ( 1 ) | ( 2 ) | ( 3 ) | ( 4 ) | ( 9 ) |
| Tasks | ( 1 ) | ( 2 ) | ( 3 ) | ( 4 ) | ( 9 ) |

General opinion :___________________________________________________________________

5th CLASS- Primary sources. (Structure of scientific articles/ Introduction to critical reading / Work on Critical Analysis of Articles)

| **5th CLASS** | **General evaluation** | | | | **SCO** |
| --- | --- | --- | --- | --- | --- |
|  | **Awful** | **Bad** | **Good** | **Great** |  |
| Methodology of the class | ( 1 ) | ( 2 ) | ( 3 ) | ( 4 ) | ( 9 ) |
| Supporting materials | ( 1 ) | ( 2 ) | ( 3 ) | ( 4 ) | ( 9 ) |
| Tasks | ( 1 ) | ( 2 ) | ( 3 ) | ( 4 ) | ( 9 ) |

General opinion :___________________________________________________________________

6th CLASS- Introduction to the work on Prescriptions

| **6th CLASS** | **GENERAL EVALUATION** | | | | **SCO** |
| --- | --- | --- | --- | --- | --- |
|  | **Awful** | **Bad** | **Good** | **Great** |  |
| Methodology of the class | ( 1 ) | ( 2 ) | ( 3 ) | ( 4 ) | ( 9 ) |
| Supporting materials | ( 1 ) | ( 2 ) | ( 3 ) | ( 4 ) | ( 9 ) |
| Tasks | ( 1 ) | ( 2 ) | ( 3 ) | ( 4 ) | ( 9 ) |

General opinion :___________________________________________________________________

**PART 5 – EVALUATION OF EACH CLASS IN MODULE II:**

7th CLASS- Pharmaceutical Attention in the World and RDC 44 legislation.

| **7th CLASS** | **GENERAL EVALUATION** | | | | **SCO** |
| --- | --- | --- | --- | --- | --- |
|  | **Awful** | **Bad** | **Good** | **Great** |  |
| Methodology of the class | ( 1 ) | ( 2 ) | ( 3 ) | ( 4 ) | ( 9 ) |
| Supporting materials | ( 1 ) | ( 2 ) | ( 3 ) | ( 4 ) | ( 9 ) |
| Tasks | ( 1 ) | ( 2 ) | ( 3 ) | ( 4 ) | ( 9 ) |

General opinion :___________________________________________________________________

8th CLASS-Dispensing medication (Analysis of videos on dispensing medication)

| **8th CLASS** | **GENERAL EVALUATION** | | | | **SCO** |
| --- | --- | --- | --- | --- | --- |
|  | **Awful** | **Bad** | **Good** | **Great** |  |
| Methodology of the class | ( 1 ) | ( 2 ) | ( 3 ) | ( 4 ) | ( 9 ) |
| Supporting materials | ( 1 ) | ( 2 ) | ( 3 ) | ( 4 ) | ( 9 ) |
| Tasks | ( 1 ) | ( 2 ) | ( 3 ) | ( 4 ) | ( 9 ) |

General opinion :___________________________________________________________________

8th CLASS-Adherence to treatment (Group intervention and creation of the script )

| **8th CLASS** | **GENERAL EVALUATION** | | | | **SCO** |
| --- | --- | --- | --- | --- | --- |
|  | **Awful** | **Bad** | **Good** | **Great** |  |
| Methodology of the class | ( 1 ) | ( 2 ) | ( 3 ) | ( 4 ) | ( 9 ) |
| Supporting materials | ( 1 ) | ( 2 ) | ( 3 ) | ( 4 ) | ( 9 ) |
| Tasks | ( 1 ) | ( 2 ) | ( 3 ) | ( 4 ) | ( 9 ) |

General opinion :___________________________________________________________________

9th CLASS - Medication errors (Tribunal)

| **9th CLASS** | **GENERAL EVALUATION** | | | | **SCO** |
| --- | --- | --- | --- | --- | --- |
|  | **Awful** | **Bad** | **Good** | **Great** |  |
| Methodology of the class | ( 1 ) | ( 2 ) | ( 3 ) | ( 4 ) | ( 9 ) |
| Supporting materials | ( 1 ) | ( 2 ) | ( 3 ) | ( 4 ) | ( 9 ) |
| Tasks | ( 1 ) | ( 2 ) | ( 3 ) | ( 4 ) | ( 9 ) |

General opinion :___________________________________________________________________

10th CLASS- System for the distribution of medication and measuring blood pressure.

| **10th CLASS** | **GENERAL EVALUATION** | | | | **SCO** |
| --- | --- | --- | --- | --- | --- |
|  | **Awful** | **Bad** | **Good** | **Great** |  |
| Methodology of the class | ( 1 ) | ( 2 ) | ( 3 ) | ( 4 ) | ( 9 ) |
| Supporting materials | ( 1 ) | ( 2 ) | ( 3 ) | ( 4 ) | ( 9 ) |
| Tasks | ( 1 ) | ( 2 ) | ( 3 ) | ( 4 ) | ( 9 ) |

General opinion :___________________________________________________________________

11th CLASS-Pharmaceutical orientation

| **11th CLASS** | **GENERAL EVALUATION** | | | | **SCO** |
| --- | --- | --- | --- | --- | --- |
|  | **Awful** | **Bad** | **Good** | **Great** |  |
| Methodology of the class | ( 1 ) | ( 2 ) | ( 3 ) | ( 4 ) | ( 9 ) |
| Supporting materials | ( 1 ) | ( 2 ) | ( 3 ) | ( 4 ) | ( 9 ) |
| Tasks | ( 1 ) | ( 2 ) | ( 3 ) | ( 4 ) | ( 9 ) |

General opinion :___________________________________________________________________

12th CLASS - Atenfar Treasure hunt

| **12th CLASS** | **GENERAL EVALUATION** | | | | **SCO** |
| --- | --- | --- | --- | --- | --- |
|  | **Awful** | **Bad** | **Good** | **Great** |  |
| Methodology of the class | ( 1 ) | ( 2 ) | ( 3 ) | ( 4 ) | ( 9 ) |
| Supporting materials | ( 1 ) | ( 2 ) | ( 3 ) | ( 4 ) | ( 9 ) |
| Tasks | ( 1 ) | ( 2 ) | ( 3 ) | ( 4 ) | ( 9 ) |

General opinion :___________________________________________________________________

13th CLASS- Orientation simulation (Recording))

| **13th CLASS** | **GENERAL EVALUATION** | | | | **SCO** |
| --- | --- | --- | --- | --- | --- |
|  | **Awful** | **Bad** | **Good** | **Great** |  |
| Methodology of the class | ( 1 ) | ( 2 ) | ( 3 ) | ( 4 ) | ( 9 ) |
| Supporting materials | ( 1 ) | ( 2 ) | ( 3 ) | ( 4 ) | ( 9 ) |
| Tasks | ( 1 ) | ( 2 ) | ( 3 ) | ( 4 ) | ( 9 ) |

General opinion :___________________________________________________________________

**PART 6- STUDENT SELF-EVALUATION:**

| **Regarding the ATENFAR II course ...** | **RANGE OF AGREEMENT** | | | | | SCO |
| --- | --- | --- | --- | --- | --- | --- |
|  | Totally disagree | Disagree | Neither agree nor disagree | Agree | Totally agree |  |
| .. I don't think I did my best. | ( 1 ) | ( 2 ) | ( 3 ) | ( 4 ) | ( 5 ) | ( 9 ) |
| ..I didn't adapt well to the technology used. | ( 1 ) | ( 2 ) | ( 3 ) | ( 4 ) | ( 5 ) | ( 9 ) |
| ...I wasn't interested in the course. | ( 1 ) | ( 2 ) | ( 3 ) | ( 4 ) | ( 5 ) | ( 9 ) |
| ... It was the first time I took a DL course. | ( 1 ) | ( 2 ) | ( 3 ) | ( 4 ) | ( 5 ) | ( 9 ) |
| ... I prepared for class by reading the suggested texts. | ( 1 ) | ( 2 ) | ( 3 ) | ( 4 ) | ( 5 ) | ( 9 ) |
| .. I paid attention and participated. | ( 1 ) | ( 2 ) | ( 3 ) | ( 4 ) | ( 5 ) | ( 9 ) |
| ... After class, I summed up what I had learnt. | ( 1 ) | ( 2 ) | ( 3 ) | ( 4 ) | ( 5 ) | ( 9 ) |
| ...I learned more from reading the texts. | ( 1 ) | ( 2 ) | ( 3 ) | ( 4 ) | ( 5 ) | ( 9 ) |
| ... I learn more from dynamic methods. | ( 1 ) | ( 2 ) | ( 3 ) | ( 4 ) | ( 5 ) | ( 9 ) |

**PART 7- SATISFACTION REGARDING PHARMACEUTICAL ATTENTION II**

| **Regarding the PHARMACEUTICAL ATTENTION II course ...** | **RANGE OF AGREEMENT** | | | | | SCO |
| --- | --- | --- | --- | --- | --- | --- |
|  | Discordo totalmente | Discordo | Nem concordo, nem discordo | Concordo | Concordo totalmente |  |
| ... I think it's better when taken in DL. | ( 1 ) | ( 2 ) | ( 3 ) | ( 4 ) | ( 5 ) | ( 9 ) |
| ... I found it well planned. | ( 1 ) | ( 2 ) | ( 3 ) | ( 4 ) | ( 5 ) | ( 9 ) |
| ... I found it stimulating. | ( 1 ) | ( 2 ) | ( 3 ) | ( 4 ) | ( 5 ) | ( 9 ) |
| ... I think it's relevant to Pharmacy studies. | ( 1 ) | ( 2 ) | ( 3 ) | ( 4 ) | ( 5 ) | ( 9 ) |
| ...The content was adequate. | ( 1 ) | ( 2 ) | ( 3 ) | ( 4 ) | ( 5 ) | ( 9 ) |
| ...It's important for my professional career. | ( 1 ) | ( 2 ) | ( 3 ) | ( 4 ) | ( 5 ) | ( 9 ) |
| ... The course objectives were attained. | ( 1 ) | ( 2 ) | ( 3 ) | ( 4 ) | ( 5 ) | ( 9 ) |

Criticism and suggestions::___________________________________________________________________________________________

_______________________________________________________________________________________________________

_______________________________________________________________________________________________________
